# Supplementary material for: Is There a Role for the Neutrophil-to-Lymphocyte Ratio for Rebleeding and Mortality Risk Prediction in Acute Variceal Bleeding? A Comparative 5-Year Retrospective Study
Source: Diseases. 2025 Aug 16;13(8):265. doi: 10.3390/diseases13080265 (PMC12385447; doi:10.3390/diseases13080265)
Supplement: Supplementary file 1 [file diseases-13-00265-s001.zip › Supplementary Table S1.pdf]

Table S1 Studies analyzing the accuracy of prognostic scores in AVB

Author	*Number of pts*	*Score, outcome, time*	*CONCLUSIONS*
CLASSICAL SCORES ONLY			
Rout G. et al, [42]	439 NVUGIB<br>572 VUGIB	GBS, mGBS, full and clinical Rockall scores (FRS, CRS), AIMS65<br>6-weeks mortality	The accuracy of prognostic scores in predicting the composite outcome (need for hospital-based intervention and 42-day mortality) was higher in NVUGIB as compared with VUGIB, AUC was 0.641 vs. 0.537 for CRS; 0.669 vs. 0.625 for FRS; 0.719 vs. 0.587 for GBS; 0.711 vs. 0.594 for mGBS; 0.567 vs. 0.548 for AIMS65.
Chandnani S.et al, [43]	141	Rockall, GBS, PNED, AIMS65<br>30-day mortality	Full Rockall, GBS, PNED, and AIMS65 performed poorly in the estimation of mortality (AUC<0.7).
Reed E. et al, [44]	71	GBS, full and clinical Rockall score (FRS, CRS)<br>In-hospital mortality	No significant differences were noted between the AUC for the GBS(0.56), FRS (0.72), and CRS (0.70).
Budimir I. et al, [47]	225	AIMS65, GBS, Rockall<br>30-days mortality	AIMS65, GBS, and full Rockall scores were similar but with low accuracy (AUC 0.70, 0.64, 0.66).
Sarwar S. et al, [51]	402	Full Rockall score<br>In-hospital mortality and rebleeding.	FRS had good predictive value for in-hospital mortality (AUC 0.834) and rebleeding (AUC 0.798).
Lu X. et al, [53]	51	AIMS65, GBS, Rockall<br>In-hospital mortality	The AUC for mortality prediction was 0.885 for AIMS65, 0.781 for GBS, and 0.767 for Rockall.
LIVER FAILURE SCORES ONLY			
Chirapongsathorn S. et al, [77]	713	New model, MELD,<br>6-weeks mortality	The 6-week overall mortality rate was 18%. New model = (1.5 * MELD score ≥18) + (3.5*shock)+ (1 * time to endoscopy < 24 h), with a cutoff value ≥ 4, had an 0.93 AUC, significantly higher than MELD score alone (0.74). Two validation analyses showed that the AUC was consistently high.
Conejo I. et al, [78]	915<br>(523 with early TIPS)	MELD, CTP score and class<br>6-weeks mortality	All 3 rules discriminated patients at high risk of death from those with low risk (early TIPS criteria 28.3% vs 7.0%; MELD19 criteria 46.0% vs 8.1%; Child C1 criteria 51.9% vs 10.9%).
Reverter E. et al, [9]	178	MELD, MELD recalibrated, CTP<br>6-weeks mortality	MELD was the best model in terms of discrimination (AUC 0.79 vs CTP 0.74, NS); it was recalibrated to predict the 6-week mortality rate with logistic regression (logit,-5.312 þ 0.207 _ MELD; bootstrapped R2, 0.3295).

Al-Freah MAB et al, [11]	177 (ICU admitted pts)	MELD, APACHE II, CTP, and NFO In-hospital mortality	The AUROC was 0.84 for MELD, 0.81 for APACHE II, 0.79 for SOFA, 0.75 for CTP, and 0.82 for NFO.
Sempere L et al, [12]	201	CTP versus MELD<br>6-week, 3- and 12-months mortality	c-STAT was better for MELD than for CTP in all timeframes: 0.804 vs 0.762 at 6W, 0.794 vs 0.760 at 3 Mo, 0.766 vs 0.741 at 12 Mo
Horvatits T. et al, [67]	472	MELD-Lactate, MELD, peak-lactate<br>30-day mortality	MELD-lactate is superior with AUC 0.82, better than MELD and peak-lactate (AUC 0.78 and 0.73).
Flores-Rendón AR et al, [52]	212	MELD, CTP. In-hospital mortality (total and bleeding-related)	Similar in-hospital total mortality for CTP and MELD (AUC 0.809 and 0.88). The bleeding-related mortality was best predicted by MELD compared to CTP (AUC 0.905 *vs* 0.794).
Elshaarawy O. et al, [57]	1517	CTP, MELD, ALBi, PALBi<br>In-hospital mortality and rebleeding.	The AUC for CTP, MELD, ALBi, and PALBi scores were 0.668, 0.689, 0.803, and 0.871. For predicting rebleeding, the AUROC for CTP, MELD, ALBI, MELD, and PALBi scores were 0.681, 0.74, 0.766, 0.769 and 0.794.
Fortune BE. et al, [58]	70	CTP, MELD<br>6-weeks mortality	AUC 0.75 for CTP, 0,79 for MELD (similar). In the calibrated model, the difference between predicted and observed mortality was significant only for MELD.
Rout G et al, [56]	285	MELD-model E, CTP-Creatinine, Augustin, D'Amico<br>6-week mortality	The MELD-E model (AUC 0.792) had the highest AUC compared to other MELD-based models (AUC 0.751), Child-Pugh score (AUC 0.737), D'Amico model (AUC 0.716) and Augustin model (AUC 0.739) In validation cohort, the discriminatory performance of MELD-E model (AUC 0.805) was higher as compared to other models including MELD score (AUC 0.771), CTP-creatinine (AUC 0.746), Augustin model (AUC 0.753) and D'Amico model (AUC 0.736).
Wong MW. et al, [61]	714	CLIF-SOFA, MELD, CTP class.<br>In-hospital and 6-weeks mortality	For in-hospital death, AUC values of CLIF-SOFA, MELD, and CTP classes were 0.964, 0.876, and 0.846. For predicting 6-week death, AUC values were 0.943, 0.817, and 0.834. CLIF-SOFA score had a higher AUC value with statistical significance.
Liu Y. et al, [62]	338	ALBi, MELD, CTP<br>1-year mortality	AUC was 0.765 for ALBi , 0.752 for CTP, 0.743 for MELD, 0.733 for MELD-Na.
Zou D. et al, [63]	631	ALBi, MELD, CTP<br>In-hospital mortality	The AUC of the ALBi, CTP, and MELD scores were 0.808, 0.785, and 0.787. In patients with hepatitis B virus-related liver cirrhosis, the AUC of the ALBI, CTP, and MELD scores were 0.865, 0.836, and 0.818.

			In patients with alcohol-related liver cirrhosis, the AUC of the ALBI, CTP, and MELD scores were 0.869, 0.860, and 0.801.
Zhong BY et al, [65]	259	ALBi, PALBi, CTP, and MELD-based nomograms and an artificial neural network (ANN) model<br>1-year rebleeding	Four nomograms showed comparable prognostic performances after internal (C-index for ALBI-, PALBI-, Child-Pugh-, and MELD-based nomograms and an artificial neural network (ANN) model: 0.879, 0.829, 0.874, and 0.798) and external (C-index: 0.720, 0.719, 0.718, and 0.703) validation.
Bae WK et al, [66]	71	Delta MELD/month, Delta CTP/month, MELD, CTP<br>6- and 12-months mortality	AUC was 0.81 for the initial CTP, 0.75 for the initial MELD, and 0.84 for DeltaCTP/month at 6 months. The AUC for DeltaMELD/month at 12 months was 0.81.
Rudler M et al, [68]	219	MELD-recalibrated, MELD<br>6-week mortality	The recalibrated MELD-based score was accurate in predicting 6-week mortality (AUC = 0.787), with better performance compared to the MELD score.
Buckholz A. et al, [69]	125 training<br>229 (validation)	MELD, MELD-3, CTP score and class.<br>6-weeks mortality	All recalibrated MELD-based and CTP-based models had excellent discrimination to identify patients at higher risk for 6-week mortality.
Xavier SA et al, [70]	111	ALBi, CTP, MELD<br>30-day and 1-year mortality	For 30-day mortality, only the ALBI score showed statistically significant results (AUC 0.80). For 1-year mortality, AUC for ALBI, CP, and MELD scores were 0.71, 0.64, and 0.66.
LIVER FAILURE VERSUS CLASSICAL PROGNOSTIC SCORES			
Motola-Kuba M. et al, [10]	160	MELD, MELD-Na, CTP, GBS, Rockall, AIMS65<br>In-hospital mortality, rebleeding	MELD best for Mortality (AUC 0.828) and AIMS65 (AUC 0.818).<br>GBS best for rebleeding (AUC 0.756) versus full Rockall (AUC 0.691)
Huy DQ et al, [13]	222	ALBi, PALBi, MELD, CTP, AIMS65<br>In-hospital mortality and rebleeding	For in-hospital mortality prediction, ALBi, PALBi, MELD and AIMS65 had good accuracy (AUC 0.81, 0.80, 0.83, and 0.82), while CTP had medium accuracy (AUC 0.79), but with no significant statistical difference.
Hassanien M et al, [14]	725<br>C-virus	CTP, Cr-CTP, MELD (including iMELD, uKELD), AIMS65<br>In-hospital mortality	AUC was 0.826 for CTP-Cr, 0.81 for CTP, 0.692 and 0.558 for MELD and iMELD, 0.527 for UKELD, and 0.525 for AIMS65.
Jamil Z. et al, [40]	217	CTP, MELD and GB score<br>In-hospital mortality	CTP was the most efficient score (AUC = 0.9, cut-off value > 7); followed by MELD (AUC = 0.8, cut-off value > 18), and GB score (AUC = 0.64, cut-off value > 14) ($p < 0.05$). The difference in CTP vs MELD was insignificant ($p > 0.05$).

			Platelets, albumin, CTP, and MELD score = independent contributors to mortality.
Sarwar S et al, [79]	366	Rockall, CTP, MELD<br>In-hospital mortality	CTP score had better c-statistics (0.801 vs. 0.688) for mortality than MELD, Rockall had 0.824.
Aluizio CLS. et al, [45]	222	Child, MELD, Rockall, GBS, AIMS65<br>6-week mortality and rebleeding.	Prediction of 6-week mortality based on Child and MELD scores were acceptable (AUC 0.72 and 0.74), but not by Rockall (AUC 0.65), GBS (AUC=0.60), and AIMS65 (AUC=0.67). No score was useful for rebleeding.
Wang F et al, [46]	202	MELD, MELD-Na, CTP, AIMS65, full Rockall score (FRS), GBS<br>6-week mortality	AUC for AIMS65 and MELD-Na (0.808 and 0.781) were superior to those of MELD (0.761), CTP (0.748), FRS (0.738), and GBS (0.726).
Mouelhi L. et al, [48]	89	CTP, MELD, MELD-Na, Rockall, GBS<br>6-weeks mortality	AUC 0.867 for MELD-Na, 0.809 for CTP, 0.777 for Rockall, 0.761 for GBS, 0.838 for MELD.
Robertson M. et al, [49]	223	AIMS65, MELD, uKELD, clinical and full Rockall score (CRS, FRS), CTP<br>In-hospital mortality	In-hospital mortality prediction by AUC was 0.84 for AIMS65, 0.81 for MELD, 0.79 for uKELD and FRS, and 0.78 for CTP and CRS.
Tantai XX. et al, [50]	330	Clinical Rockall score (CRS), AIMS65, GBS, mGBS, CANUKA, CTP, MELD, MELD-Na<br>In-hospital mortality, rebleeding	For in-hospital mortality, the CRS, CTP, AIMS65, MELD-Na, and MELD showed excellent discriminative ability (AUC > 0.8). The AUC of the mGBS, CANUKA, and GBS were relatively small, but clinically acceptable (>0.7). For rebleeding, the discriminative ability of the CTP and CRS were clinically acceptable, with AUC of 0.717 and 0.716.
Mohammad AN. et al, [54]	120	AIMS65, SOFA, MELD, APACHE II, CTP<br>In-hospital mortality	Best AUC for AIMS65 (0.97) and SOFA (0.96), followed by MELD (0.88), APACHEII score (0.86), and CTP score (0.82). AIMS65 score showed the best sensitivity, specificity, and negative and positive predictive values.
Lee JY et al, [56]	136	CTP, MELD, Rockall<br>6-week and 1-year mortality	The c-statistics of CTP, MELD, and Rockall for 6-week mortality rate were 0.809, 0.804, and 0.787. For the 1-year mortality rate, c-statistics 0.765, 0.780, and 0.730.
Iino C et al, [60]	47	MELD, CTP, GBS<br>1-week and 6-week mortality	For 1-week mortality,GBS was superior to MELD and CTP (AUC 0.82,0.82,and 0.71). For 6-week mortality,MELD was superior to CTP and GBS (AUC 0.83,0.69,and 0.67).
Jin Yan et al, [55]	110	MELD, AIMS65<br>6-week mortality	The AUC for MELD was 0.813, and 0.825 for AIMS65 (not statistically significant).
NLR studies			

Chen X., et al [18]	193	NLR, PLR<br>Only rebleeding risk	NLR and PLR were significantly increased in rebleeding patients with AVB compared with the nonrebleeding patients ; AUC of NLR and PLR was 0.7037 and 0.7468.
Bai Z. et al, [80]	865 training 817 validation	CAGIB, MELD, MELD-Na, NLR, CTP<br>In-hospital mortality	In the training cohort, the AUC of CAGIB score for predicting in-hospital death was 0.829, higher than CTP (0.762), MELD (0.778), and NLR (0.587) scores.<br>In the validation cohort, the AUC of the CAGIB score (0.714), remained higher than that of CTP (0.693), MELD (0.662), MELD-Na (0.660), and NLR (0.538) scores.
Zhao Y. et al, [64]	379	CAGIB, MELD, CTP, NLR<br>In-hospital mortality	In HCC patients, the AUCs of CTP, CAGIB, MELD, and NLR scores were 0.842, 0.840, 0.798, and 0.688. In patients without HCC, the AUCs of CTP, CAGIB, MELD, and NLR scores were 0.864, 0.780, 0.800, and 0.747.
Zhang L et al [38]	57 peptic ulcer bleeding, 33 AVB	NLR, PLR<br>Differentiation NVUGIB from AVB	AUCs in distinguishing PUB from EGVB were 0.859, 0.811, 0.760, 0.952, and 0.687 for leukocyte, neutrophil, lymphocyte, platelet, and PLR. NLR had no statistical significance ($p > 0.05$).
Chen L. et al [39]	726	NLR, GBS, full RS, combinations NLR-GBS and NLR-FRS<br><br>In-hospital mortality, rebleeding, transfusions	High level of NLR = risk factor for death in patients with UGIB ($p = 0.028$). NLR, GBS, FRS, NLR-GBS, and NLR-FRS have sufficient accuracy in predicting inpatient mortality, endoscopic treatment, and transfusion treatment. The AUC was 0.7037 for NLR. In the comprehensive prediction of adverse outcomes, NLR-GBS has the highest AUROC (0.739), and in predicting inpatient mortality, NLR-FRS has the highest AUROC (0.763).

77. Chirapongsathorn S, Akkarachinores K, Chaiprasert A. Development and validation of prognostic model to predict mortality among cirrhotic patients with acute variceal bleeding: A retrospective study. JGH Open. 2021 May 5;5(6):658-663. doi: 10.1002/jgh3.12550. PMID: 34124382; PMCID: PMC8171152.

78. Conejo I, Guardascione MA, Tandon P, Cachero A, Castellote J, Abraldes JG, Amitrano L, Genescà J, Augustin S. Multicenter External Validation of Risk Stratification Criteria for Patients With Variceal Bleeding. Clin Gastroenterol Hepatol. 2018 Jan;16(1):132-139.e8. doi: 10.1016/j.cgh.2017.04.042. Epub 2017 May 10. PMID: 28501536.

79. Sarwar S, Khan AA, Tarique S. Comparison of MELD, Child Pugh score and Rockall score for predicting rebleeding and in-hospital mortality in patients of variceal bleeding. J Coll Physicians Surg Pak. 2008 Aug;18(8):524-5. PMID: 18798596.

80. Bai Z, Li B, Lin S, Liu B, Li Y, Zhu Q, Wu Y, Yang Y, Tang S, Meng F, Chen Y, Yuan S, Shao L, Qi X. Development and Validation of CAGIB Score for Evaluating the Prognosis of Cirrhosis with Acute Gastrointestinal Bleeding: A Retrospective Multicenter Study. Adv Ther. 2019 Nov;36(11):3211-3220. doi: 10.1007/s12325-019-01083-5. Epub 2019 Sep 11. PMID: 31512140; PMCID: PMC6822790.
