# Supplementary material for: Is There a Role for the Neutrophil-to-Lymphocyte Ratio for Rebleeding and Mortality Risk Prediction in Acute Variceal Bleeding? A Comparative 5-Year Retrospective Study
Source: Diseases. 2025 Aug 16;13(8):265. doi: 10.3390/diseases13080265 (PMC12385447; doi:10.3390/diseases13080265)
Supplement: Supplementary file 1 [file diseases-13-00265-s001.zip › Supplementary Table S2-S7.pdf]

**Table S2** Rockall score [7]	*0*	*1*	*2*	*3*
Age (CRS, FRS)	<60	60-79	≥80	-
Shock (CRS, FRS)	P<100<br>sBP≥100	P≥100<br>≥100	sBP<100	-
Comorbidities (CRS, FRS)	NO major	-	Cardiac failure, coronary ischemia	Renal/liver failure<br>Disseminated malignancy
Diagnosis (FRS)	MW<br>No lesion<br>No stigmata	Other exc. malignancy	Malignancy	
Bleeding stigmata (FRS)	No/dark spot	-	Blood, adherent clot<br>Visible/spurting vessel	-

P=pulse, sBP=systolic Blood Pressure, MW=Mallory-Weiss syndrome, CRS =clinical Rockall score, FRS=full Rockall score

**Table S3** Glasgow-Blatchford score [7]		
Urea (mg/dl)	39-47	2
	48-60	3
	60-149	4
	≥150	6
Hb (g/dl)	Men 12-12.99	1
	Men ≥10	3
	Woman ≥10	1
	Both sexes <10	6
Systolic blood pressure	100-109	1
(mm Hg)	90-99	2
	<90	3
Pulse (>100/min)		1
Melena		1
Syncope		2
Liver disease		2
Cardiac failure		2

sBP=systolic blood pressure

Table S4 Modified Glasgow-Blatchford score [7]

Urea (mg/dl)	39-47	2
	48-60	3
	60-149	4
	≥150	6
Hb (g/dl)	Men 12-12.99	1
	Men ≥10	3
	Woman ≥10	1
	Both sexes <10	6
Systolic blood pressure (mm Hg)	100-109	1
	90-99	2
	<90	3
Pulse (>100/min)		1

**Table S5** AIMS65 score [7]	*Score*
Age>65 yr	1
Systolic blood pressure<90	1
Altered mental status	1
Alb<3 g%	1
INR>1,5	1

**Table S6** Child-Turcotte-Pugh (CTP) and CTP-creatinine [7]		
Ascites	Absent	1
	Mild	2
	Large	3
Encephalopathy	Absent	1
	Grade 1-2	2
	Grade 3-4	3
Serum albumin	>3.5	1
(g/dl)	2.8-3.5	2
	<2.8	3
Total bilirubin (mg/dl)	<2	1
	2-3	2
	>3	3
INR	<1.7	1
	1.7-2.3	2
	>2.3	3
Creatinine (mg/dl)	<1.3	0
-only for CTP-creatinine	1.3-.18	2
	>1.8	4

Table S7 MELD, MELD-3, MELD-Na, UKELD, ALBI, PALBi scores

MELD=9.57 * ln(creatinine) + 3.78 * ln(BR) + 11.20 * ln(INR) + 6.43, rounded to the nearest integer [10,13,40,41].

MELD-3 =1.33 (if female) + 4.56∗$\log_e$ (BR) + 0.82∗(137–Na) – 0.24∗(137-Na)∗$\log_e$ (BR) + 9.09∗$\log_e$ (INR) + 11.14∗$\log_e$ (creatinine) + 1.85∗(3.5–albumin) – 1.83∗(3.5–albumin)∗$\log_e$ (creatinine) + 6 [41].

MELD-Na = MELD+1.32* (137−Na) − 0.033 * MELD* (137−Na), rounded to the nearest integer, Na bound between 125 and 137 mmol/L.

UKELD =5 × {1.5 × $\log_e$(INR) + 0.3 × $\log_e$(creatinine, μmol/l) + 0.6 × $\log_e$(BR, μmol/l) − 13 × $\log_e$(Na, mmol/l) + 70}, rounded to the nearest integer [14].

ALBi = 0.66 × log10(BR, μmol/L) − 0.085(albumin, g/L)

PALBi = 2.02 × $\log_{10}$BR– 0.37 × ($\log_{10}$BR) – 0.04 × ALB– 3.48 × $\log_{10}$PLT + 1.01 ×($\log_{10}$PLT) [13].

BR=total bilirubin, ALB=serum albumin, PLT=Platelets

References:

7. Cazacu SM, Alexandru DO, Statie RC, Iordache S, Ungureanu BS, Iovănescu VF, Popa P, Sacerdoțianu VM, Neagoe CD, Florescu MM. The Accuracy of Pre-Endoscopic Scores for Mortality Prediction in Patients with Upper GI Bleeding and No Endoscopy Performed. Diagnostics (Basel). 2023 Mar 21;13(6):1188. doi: 10.3390/diagnostics13061188.

10. Motola-Kuba M, Escobedo-Arzate A, Tellez-Avila F, Altamirano J, Aguilar-Olivos N, González-Angulo A, Zamarripa-Dorsey F, Uribe M, Chávez-Tapia NC. Validation of prognostic scores for clinical outcomes in cirrhotic patients with acute variceal bleeding. Ann Hepatol. 2016 Nov-Dec 2016;15(6):895-901. doi: 10.5604/16652681.1222107.

13. Huy, D.Q.; Chung, N.V.; Dong, D.T. Value of Some Scoring Systems for the Prognosis of Rebleeding and In-Hospital Mortality in Liver Cirrhosis with Acute Variceal Bleeding. Gastroenterol. Insights 2023, 14, 144-155. https://doi.org/10.3390/gastroent14020011.

14. Hassanien, M.; El-Ghannam, M.; El-Talkawy, M.D.; Abdelrahman, Y.; El Attar, G.; Abu Taleb, H. Risk Scoring Systems to Predict In-Hospital Mortality in Patients with Acute Variceal Bleeding due to Hepatitis C Virus induced Liver Cirrhosis. Gastroenterol. Insights 2018, 9, 7629. https://doi.org/10.4081/gi.2018.7629

40. Jamil Z, Perveen S, Khalid S, Aljuaid M, Shahzad M, Ahmad B, Waheed Y. Child-Pugh Score, MELD Score and Glasgow Blatchford Score to Predict the In-Hospital Outcome of Portal Hypertensive Patients Presenting with Upper Gastrointestinal Bleeding: An Experience from Tertiary Healthcare System. J Clin Med. 2022 Nov 9;11(22):6654. doi: 10.3390/jcm11226654.

41. Kim WR, Mannalithara A, Heimbach JK, Kamath PS, Asrani SK, Biggins SW, Wood NL, Gentry SE, Kwong AJ. MELD 3.0: The Model for End-Stage Liver Disease Updated for the Modern Era. Gastroenterology. 2021 Dec;161(6):1887-1895.e4. doi: 10.1053/j.gastro.2021.08.050.
